# Procalcitonin Elevation in Intoxication- and Immobilization-Related Non-Traumatic Rhabdomyolysis: Association with Muscle Injury Severity and Renal Dysfunction

**DOI:** 10.3390/biomedicines14051085

**Published:** 2026-05-11

**Authors:** Linas Zdanavičius, Gabija Laubner Sakalauskienė, Viktorija Antonova Šiaudvytė, Robertas Badaras

**Affiliations:** 1 Clinic of Emergency Medicine, Faculty of Medicine, Vilnius University, LT-03101 Vilnius, Lithuania; 2 Faculty of Medicine, Vilnius University, LT-03101 Vilnius, Lithuania; gabija.laubner@mf.vu.lt (G.L.S.); robertas.badaras@mf.vu.lt (R.B.); 3 Republican Vilnius University Hospital, LT-04130 Vilnius, Lithuania; viktorija.siaudvyte@rvul.lt

**Keywords:** procalcitonin, non-traumatic rhabdomyolysis, creatine kinase

## Abstract

**Background:** Procalcitonin (PCT) is widely used as a biomarker of bacterial infection, but it may also increase in non-infectious inflammatory states. This study investigated the association of PCT with muscle injury severity and renal dysfunction in patients with non-traumatic rhabdomyolysis. **Methods:** We prospectively enrolled 39 adults with non-traumatic rhabdomyolysis (n = 39). Admission and longitudinal laboratory data included creatine kinase (CK), myoglobin, PCT, C-reactive protein (CRP), creatinine, and urea. Associations were assessed using Spearman correlation analysis with false-discovery-rate correction for multiple comparisons. Independent predictors of admission PCT were evaluated using linear regression with PCT (log10) as the dependent variable. Longitudinal dynamics were analyzed using linear mixed-effects models and a generalized mixed-effects sensitivity model. **Results:** At admission, after false-discovery-rate correction, PCT remained positively correlated with creatinine, CK, urea, and CRP; the weaker correlation with myoglobin did not remain significant after correction. In the primary admission model, both creatinine (log10) and CK (log10) were independently associated with PCT (log10), whereas infection status was not. In the broader sensitivity model, only creatinine (log10) remained significant. Longitudinal analyses showed that CK (log10) remained independently associated with PCT over time, including in the gamma mixed-effects sensitivity model; in that model, infection status was not independently associated with PCT. **Conclusions:** In this exploratory single-centre cohort of patients with predominantly intoxication- or immobilization-related “found-down” non-traumatic rhabdomyolysis, PCT elevation was associated with markers of muscle injury and renal dysfunction. Infection status was not independently associated with PCT in the fitted models; however, the modest sample size means that smaller infection-related effects cannot be excluded. These findings should therefore be interpreted cautiously and require confirmation in larger, more etiologically diverse rhabdomyolysis cohorts.

## 1. Introduction

Rhabdomyolysis (RM) is an acute syndrome caused by skeletal-muscle injury with release of intracellular contents into the bloodstream [1,2,3,4]. Non-traumatic RM [1,2,3]—often seen in “found-down” presentations after intoxication [2,3,4,5,6,7,8,9], prolonged immobilization [1,2,3,5,7,8,10], or metabolic derangements [2,7,11]—is common in emergency and critical-care settings [2,7,12] and may lead to acute kidney injury [1,2,3,6,7,13,14,15,16,17,18]. Creatine kinase (CK) and myoglobin are standard markers of muscle damage [1,2,3,6,11,16,17,18,19,20], but their relationship to systemic inflammatory biomarkers remains an active area of investigation [21,22,23].

Procalcitonin (PCT) is widely used to support the diagnosis of severe bacterial infection and guide antimicrobial therapy [21,24,25,26,27]. However, PCT can rise in the absence of infection [21] in conditions characterized by extensive sterile inflammation (e.g., major trauma, surgery, burns, heat illness) [21,25,28]. Reports describing markedly elevated PCT in RM without microbiological evidence of infection [21,28,29] have raised questions about the specificity of PCT in this context and created uncertainty for clinicians deciding whether an elevated PCT necessarily implies bacterial infection [30,31].

Biologically, several mechanisms plausibly link muscle injury to increased PCT in the absence of pathogens [21,25,27,32]. Necrotic and damaged myocytes release damage-associated molecular patterns (DAMPs) that activate pattern-recognition receptors (e.g., Toll-like receptors) on immune and parenchymal cells, triggering release of pro-inflammatory cytokines such as TNF-α, IL-1β, and IL-6 [21,24,33]. These cytokines upregulate extra-thyroidal PCT production [21,27] (notably in liver, lung, and adipose tissue) [21,24], producing a systemic PCT surge that can mimic the biochemical profile of sepsis [21]. PCT may also rise earlier than CRP because it responds more directly to IL-1/TNF, whereas CRP synthesis is predominantly IL-6-driven [21,24]. Organ dysfunction can contribute modestly—renal failure may elevate baseline PCT—but current evidence suggests that increased production, not impaired clearance, is the dominant driver in severe sterile inflammation [24,27,28].

Despite these observations, the literature remains heterogeneous and sometimes divergent: some studies emphasize PCT’s utility for infection triage in critical illness [34,35], while others document substantial elevations during non-infectious tissue injury [32,36]. High-quality data that quantify how strongly PCT tracks the extent of muscle injury—independent of infection—are limited [32,36,37], and this gap has practical implications for antibiotic stewardship and diagnostic decision-making in RM.

## 2. Materials and Methods

This prospective observational study included 39 consecutive adult patients admitted to the Toxicology Centre of the Republican Vilnius University Hospital, Lithuania, with intoxication- or prolonged immobilization-related non-traumatic rhabdomyolysis. The study protocol was approved by the Vilnius Regional Committee on Biomedical Research Ethics (license No. 2023/10-1534-1008).

Patients were eligible for inclusion if they presented with a clinical history compatible with prolonged immobilization or intoxication-related loss of consciousness (the so-called “found-down” presentation) and showed laboratory evidence of skeletal muscle injury, defined by serum creatine kinase (CK) and myoglobin concentrations at least twice above the upper reference limit (normal CK 0–190 U/L, myoglobin <72 µg/L). This relatively low threshold was chosen to avoid missing early or evolving cases of rhabdomyolysis, since CK and myoglobin exhibit delayed pharmacokinetics—both may rise gradually and peak several hours to days after the initial muscle injury [38]. Including patients with moderate early elevations therefore ensured enrollment of those in whom biomarker concentrations could subsequently increase during hospitalization.

The diagnosis of rhabdomyolysis was based on the clinical judgment of the admitting physician, integrating the patient’s history, physical findings, and laboratory results. CK testing was typically performed when rhabdomyolysis was clinically suspected, most often in patients presenting after alcohol or substance intoxication or after being found unconscious for prolonged periods. Because non-traumatic rhabdomyolysis may occur without elevated compartment pressures [2,16], pressure measurements were not required. Patients with traumatic crush injuries, high-energy trauma, or other alternative causes of CK elevation such as recent surgery, seizures, myocardial infarction, or vascular occlusion were excluded from the study.

At admission, all participants underwent laboratory testing that included CK, myoglobin, procalcitonin (PCT), C-reactive protein (CRP), urea, and creatinine. According to the study protocol, these parameters were subsequently repeated daily for the first 7 days and again on days 10 and 14, or until hospital discharge. Demographic data (age and sex) and infection status were also recorded. Available medical records were reviewed for pre-existing chronic kidney disease. None of the enrolled patients had documented chronic kidney disease before admission. In accordance with hospital policy, all patients were screened for common viral pathogens, including SARS-CoV-2 and influenza, at admission. Routine viral serology for hepatitis B virus (HBV), hepatitis C virus (HCV), and human immunodeficiency virus (HIV) was also performed in all patients, and all results were negative. None of the participants exhibited clinical features of viral illness during hospitalization; therefore, viral infections were considered unlikely to contribute to biomarker changes.

Bacterial infection was defined as either microbiological confirmation from blood, urine, or respiratory cultures, or strong clinical or radiological evidence consistent with infection. The latter included new or progressive pulmonary infiltrates with compatible symptoms (fever, productive cough, elevated inflammatory markers), urinalysis showing bacteriuria accompanied by leukocyturia and positive nitrite tests, or the presence of localized signs of infection such as cellulitis with erythema and purulent discharge. In addition, patients were classified as infected if they had persistent fever >38.5 °C associated with leukocytosis or a left shift and were started on systemic antibiotic therapy based on a documented clinical assessment by the treating physician. In patients classified as non-infected, available cultures, when obtained, were negative or not clinically suggestive of infection, and no subsequent clinical course during hospitalization supported occult bacterial infection. Each case was independently reviewed by two investigators (L.Z. and R.B.) who were blinded to biomarker results when determining infection status. Any discrepancies were resolved by consensus after full review of all clinical, radiological, and microbiological data. This two-step adjudication process ensured consistent classification of patients as infected or non-infected. Of the 39 patients, 19 (48.7%) were classified as infected and 20 (51.3%) as non-infected.

Descriptive statistics were used to summarize admission characteristics. The distribution of continuous variables was assessed using the Shapiro–Wilk test. Because most biomarkers were right-skewed, admission comparisons were summarized descriptively, pairwise associations were analyzed using Spearman’s correlation coefficients, and biomarkers entered into regression models were log10-transformed as appropriate. For the correlation matrix, *p*-values were adjusted for multiple comparisons using the Benjamini–Hochberg false-discovery-rate procedure across all 21 tested pairwise correlations.

To identify independent predictors of admission PCT concentrations, multiple linear regression was performed with PCT (log10) as the dependent variable. The primary admission model included CK (log10), creatinine (log10), and infection status as predictors. Urea was analyzed descriptively and in correlation analyses as an additional marker of renal dysfunction, but was not entered into the main multivariable models because it overlapped biologically with creatinine, had slightly fewer available observations, and creatinine was selected a priori as the more direct renal function marker for parsimonious modeling. A broader sensitivity model additionally included age, sex, CRP (log10), myoglobin (log10), and a sex × infection interaction term. Model adequacy was assessed using residual normality testing, Breusch–Pagan and Levene tests, and variance-inflation factors. Because residual diagnostics suggested some violation of model assumptions, inferential statistics for the admission models were based on heteroscedasticity-robust standard errors. In addition, bootstrap bias-corrected and accelerated (BCa) 95% confidence intervals based on 1000 resamples were calculated to assess the robustness of the regression results.

Because severe rhabdomyolysis may itself produce fever, leukocytosis, and other systemic inflammatory features, we performed an additional sensitivity analysis using a stricter definition of infection based solely on microbiologically confirmed positive cultures. In this analysis, patients with culture-proven infection were compared with all remaining patients. Admission PCT was compared using the Mann–Whitney U test, and an exploratory multivariable linear regression model was fitted with PCT (log10) as the dependent variable and culture-proven infection, creatinine (log10), and CK (log10) as predictors. The sensitivity regression model using microbiologically proven infection was estimated using the same inferential approach as the primary admission model, with heteroscedasticity-robust standard errors and bootstrap bias-corrected and accelerated (BCa) 95% confidence intervals based on 1000 resamples.

Longitudinal biomarker dynamics were analyzed using a repeated-measures dataset containing all available daily observations obtained during hospitalization, for up to 14 days or until discharge. A linear mixed-effects model with patient identification number as a random intercept was fitted with PCT (log10) as the dependent variable and age and CK (log10) as fixed effects. To address temporal effects, hospital day was added as a fixed effect in an additional time-adjusted mixed-effects model. A CK (log10) × hospital day interaction was also tested to evaluate whether the association between CK and PCT changed during hospitalization. Because PCT remained markedly right-skewed over time, a generalized mixed-effects model with gamma distribution and log link was additionally fitted as a distributional sensitivity analysis, including CK (log10), age, and infection status as fixed effects and patient identification number as a random intercept.

No formal a priori sample size or power calculation was performed. The study was designed as an exploratory prospective observational analysis of consecutively enrolled patients with a relatively uncommon clinical presentation at a single centre. Accordingly, the analyses were intended primarily to estimate associations and generate hypotheses rather than to exclude modest independent effects of infection on PCT. As an approximate post-hoc sensitivity consideration, with 39 patients and three predictors in the primary admission model, the analysis would have approximately 80% power at α = 0.05 only to detect a moderate-to-large incremental effect of an individual predictor, corresponding to Cohen’s f^2^ of approximately 0.21. Therefore, smaller independent effects of infection on PCT could have remained undetected.

Statistical significance was set at *p* < 0.05 (two-tailed). All analyses were conducted using jamovi (version 2.7, The jamovi project, 2025).

## 3. Results

### 3.1. Baseline Characteristics

The admission dataset comprised 39 patients, including 31 men (79.5%) and 8 women (20.5%). Nineteen patients (48.7%) met the study criteria for bacterial infection. Baseline characteristics stratified by infection status are presented in Table 1.

The mean age of the cohort was 47.8 ± 17.1 years. Patients classified as infected were older than those without infection (53.4 ± 19.5 vs. 42.5 ± 12.8 years, *p* = 0.043). No statistically significant between-group differences were observed for sex distribution or admission concentrations of procalcitonin (PCT), creatine kinase (CK), myoglobin, C-reactive protein (CRP), creatinine, or urea. Of the 19 patients classified as infected, 10 (52.6%) had microbiologically positive cultures, corresponding to 25.6% of the full cohort.

### 3.2. Correlation Analysis

Spearman correlation analysis of admission biomarkers and age is shown in Table 2. After Benjamini–Hochberg false-discovery-rate correction across 21 pairwise correlations, PCT remained positively correlated with creatinine (ρ = 0.587, adjusted *p* < 0.001), urea (ρ = 0.528, adjusted *p* = 0.004), CRP (ρ = 0.501, adjusted *p* = 0.004), and CK (ρ = 0.486, adjusted *p* = 0.005). The weaker association between PCT and myoglobin did not remain significant after correction (ρ = 0.326, adjusted *p* = 0.082). CK was strongly correlated with myoglobin (ρ = 0.736, adjusted *p* < 0.001), and CRP was correlated with both urea (ρ = 0.528, adjusted *p* = 0.004) and CK (ρ = 0.393, adjusted *p* = 0.036). Creatinine was also correlated with urea (ρ = 0.541, adjusted *p* = 0.004). Correlations between age and admission biomarkers did not remain significant after false-discovery-rate correction.

Overall, these admission correlations suggested that higher PCT concentrations were associated with greater muscle injury and renal dysfunction, while also showing a moderate relationship with systemic inflammation.

### 3.3. Admission Multivariable Regression Analysis

To identify independent predictors of admission PCT, linear regression models were fitted using PCT (log10) as the dependent variable.

In the primary model, creatinine (log10), infection status, and CK (log10) were entered as predictors. Both creatinine (log10) (estimate 1.304, standardized β = 0.470, 95% CI 0.432 to 2.057, *p* = 0.014) and CK (log10) (estimate 0.447, standardized β = 0.382, 95% CI 0.152 to 0.767, *p* = 0.009) were independently associated with PCT (log10), whereas infection status was not (estimate 0.106, 95% CI −0.381 to 0.591, *p* = 0.700). This model explained 45.6% of the variance in PCT (log10) (R^2^ = 0.456; adjusted R^2^ = 0.409; F = 9.77; *p* < 0.001). Collinearity was low, with variance inflation factor values between 1.03 and 1.08.

Residual diagnostics indicated some instability of model assumptions, including residual non-normality and evidence of unequal variance across groups. Therefore, inferential statistics were based on heteroscedasticity-robust standard errors, and bootstrap BCa confidence intervals were additionally examined. The bootstrap analysis supported the same overall interpretation, with confidence intervals for creatinine (log10) and CK (log10) remaining above zero, whereas the interval for infection status included zero.

In a broader sensitivity model additionally including age, sex, CRP (log10), myoglobin (log10), and a sex × infection interaction term, overall model fit increased (R^2^ = 0.553; adjusted R^2^ = 0.434; F = 4.64; *p* < 0.001), but only creatinine (log10) remained independently associated with PCT (log10) (estimate 1.212, standardized β = 0.437, 95% CI 0.428 to 1.997, *p* = 0.004). CRP (log10) showed a borderline association (estimate 0.363, *p* = 0.071), whereas CK (log10), myoglobin (log10), age, sex, infection status, and the interaction term were not statistically significant.

**Figure A1:**
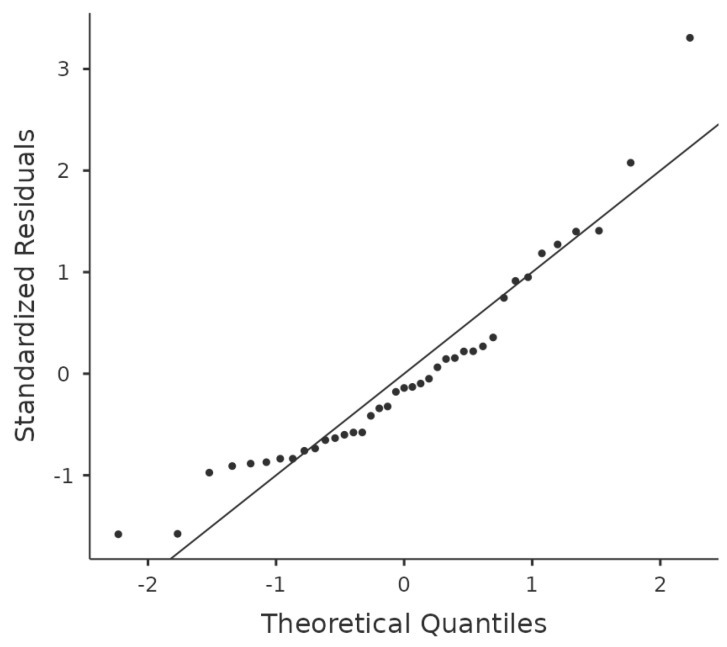
Q-Q plot of standardized residuals.

**Figure A2:**
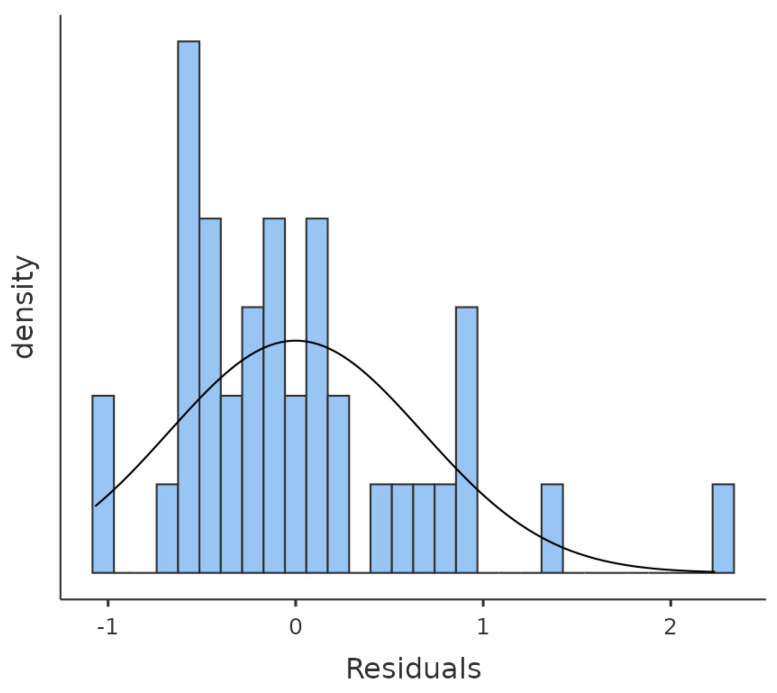
Histogram of residuals.

**Figure A3:**
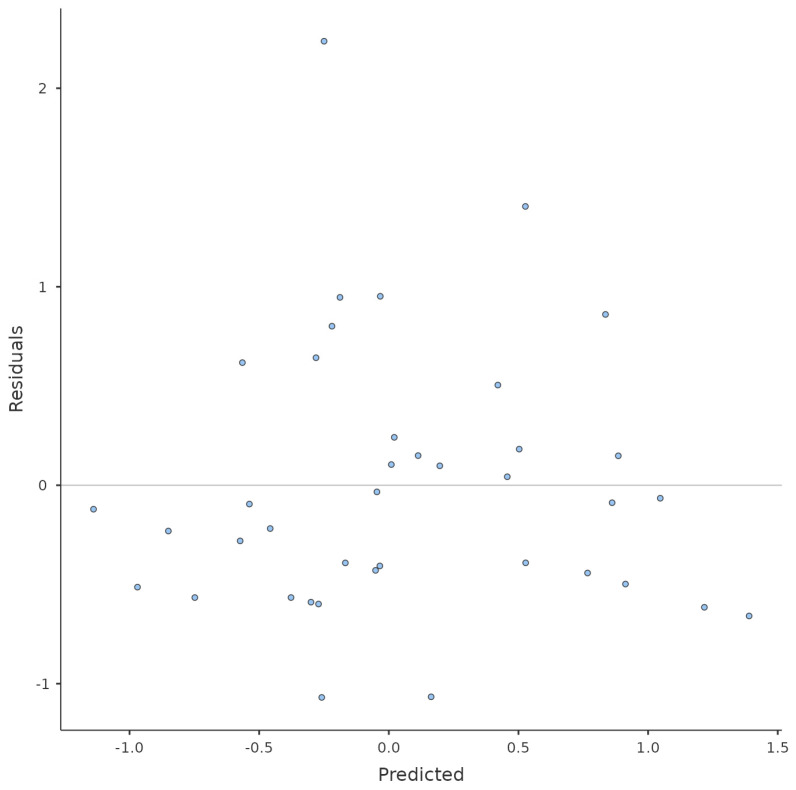
Residuals-versus-predicted values plot.

Diagnostic plots for the primary admission regression model are provided in Appendix A, Figure A1, Figure A2 and Figure A3. The Q-Q plot and residual histogram demonstrated residual non-normality, while the residuals-versus-predicted values plot was inspected to assess homoscedasticity and overall model fit. These observations, together with formal diagnostic testing, supported the use of heteroscedasticity-robust standard errors and bootstrap BCa confidence intervals.

### 3.4. Sensitivity Analysis Using Microbiologically Proven Infection

To address the possibility of incorporation bias arising from the clinical adjudication of infection, a sensitivity analysis was performed using a stricter definition restricted to microbiologically confirmed infection. Ten of 39 patients (25.6%) had positive microbiological cultures. Admission PCT concentrations were similar between patients with culture-proven infection and all remaining patients (median 1.97 vs. 1.37 ng/mL; Mann–Whitney U = 135, *p* = 0.748).

Using the same robust and bootstrap-based inferential approach as in the primary admission model, culture-proven infection was not independently associated with PCT (log10) (estimate −0.030, 95% CI −0.569 to 0.590, *p* = 0.916). In contrast, both creatinine (log10) (estimate 1.337, 95% CI 0.340 to 2.055, *p* = 0.003) and CK (log10) (estimate 0.442, 95% CI 0.177 to 0.774, *p* = 0.003) remained independently associated with PCT (log10). The model explained 45.3% of the variance in PCT (log10) (R^2^ = 0.453; adjusted R^2^ = 0.406; *p* < 0.001). 

### 3.5. Longitudinal Mixed-Effects Analysis

Longitudinal analysis included 143 repeated measurements obtained from 39 patients during hospitalization.

In the primary linear mixed-effects model, CK (log10) remained independently associated with PCT (log10) over time (estimate 0.320, 95% CI 0.222 to 0.418, *p* < 0.001), whereas age was not significantly associated with PCT (*p* = 0.765). The marginal R^2^ was 0.123 and the conditional R^2^ was 0.796, with an intraclass correlation coefficient of 0.767, indicating substantial clustering of repeated observations within patients.

In a time-adjusted sensitivity model including hospital day as a fixed effect, CK (log10) remained independently associated with PCT (log10) (estimate 0.228, 95% CI 0.071 to 0.384, *p* = 0.005), whereas hospital day was not significantly associated with PCT (estimate −0.040, 95% CI −0.093 to 0.014, *p* = 0.143). A CK (log10) × hospital day interaction was also tested and was statistically significant (estimate −0.056, 95% CI −0.090 to −0.022, *p* = 0.002), suggesting that the CK–PCT association was strongest earlier during hospitalization and attenuated over time.

Because longitudinal PCT values remained right-skewed, a generalized mixed-effects model with gamma distribution and log link was fitted as a sensitivity analysis. In this model, CK again remained strongly associated with PCT (estimate 0.789, 95% CI 0.604 to 0.973, *p* < 0.001), whereas neither infection status (estimate 0.981, 95% CI −0.266 to 2.228, *p* = 0.122) nor age was independently associated (*p* = 0.794). The gamma model showed a marginal R^2^ of 0.191 and a conditional R^2^ of 0.853, with an intraclass correlation coefficient of 0.819.

Taken together, the longitudinal analyses supported a persistent association between CK and PCT across hospitalization. The time-adjusted analysis indicated that this association was strongest earlier during hospitalization and weakened over time, while infection status was not independently associated with PCT in the gamma mixed-effects sensitivity model.

### 3.6. Summary of Findings

Overall, admission and longitudinal analyses showed that PCT was associated with renal dysfunction and muscle injury markers in this “found-down” non-traumatic rhabdomyolysis cohort. Creatinine was the most robust admission predictor, while CK was associated with PCT in the primary admission model, microbiology-only sensitivity model, and longitudinal analyses, although not in the broader admission sensitivity model. Infection status was not independently associated with PCT in the fitted models, but this finding remains inconclusive because of the modest sample size.

## 4. Discussion

In this exploratory analysis of 39 patients with intoxication- or immobilization-related non-traumatic rhabdomyolysis, PCT elevation was associated with markers of muscle injury and renal dysfunction, while its relationship with infection status was less consistent.

At admission, PCT correlated with CK, creatinine, urea, and CRP after false-discovery-rate correction. The weaker unadjusted association between PCT and myoglobin did not remain significant after correction. In the primary admission regression model, both CK and creatinine were independently associated with PCT, whereas infection status was not. In the broader sensitivity model, only creatinine remained independently associated with PCT, while the CK effect was attenuated after inclusion of CRP and myoglobin. This suggests that creatinine was the most robust admission predictor, whereas the independent association between CK and PCT partly overlapped with other markers of muscle injury and systemic inflammation. Nevertheless, CK remained associated with PCT in the primary admission model, the microbiology-only sensitivity model, and the longitudinal mixed-effects analyses.

Because severe rhabdomyolysis can itself produce fever, leukocytosis, and a sterile systemic inflammatory response that mimics sepsis [4,21,32,39], infection classification based partly on clinical and radiological features may be susceptible to misclassification or incorporation bias. To address this, we performed a sensitivity analysis restricted to microbiologically proven infection. Culture-proven infection was not independently associated with PCT after adjustment for CK and creatinine, whereas both CK and creatinine remained significant predictors. However, this analysis included only 10 culture-positive patients and should therefore be interpreted as a high-specificity sensitivity analysis, not as definitive evidence excluding an infection-related PCT effect. A strict culture-only definition may also misclassify clinically relevant culture-negative infections as non-infected, particularly in pneumonia, aspiration-related infection, cellulitis, or partially treated infection.

The longitudinal analyses further supported an association between muscle injury and PCT. Across 143 repeated measurements, CK remained independently associated with PCT in the primary linear mixed-effects model and after adjustment for hospital day. The significant CK × hospital day interaction suggested that the CK–PCT association was strongest early during hospitalization and weakened over time, consistent with changing biomarker kinetics during clinical recovery. In the gamma mixed-effects sensitivity model, CK again remained associated with PCT, whereas infection status did not reach statistical significance. Taken together, these findings suggest that renal dysfunction contributes particularly to admission PCT elevation, while longitudinal PCT trajectories are influenced by the severity and timing of muscle injury.

These findings are clinically relevant because PCT is frequently used as a biomarker of bacterial infection or sepsis, but severe muscle breakdown and renal impairment may reduce its specificity in this setting. The present results should not be extrapolated to the full spectrum of non-traumatic rhabdomyolysis, including exertional, metabolic, endocrine, seizure-related, medication-induced, autoimmune, or primarily infectious etiologies. Rather, they apply most directly to patients presenting after intoxication-related loss of consciousness or prolonged immobilization, in whom sterile inflammation, muscle necrosis, renal dysfunction, and possible infection may coexist. Previous studies have mainly evaluated PCT as a marker of sepsis or bacterial infection [34,35], while other reports have described PCT elevation in non-infectious tissue injury states, including trauma, cardiac arrest, heatstroke, and rhabdomyolysis [6,25,32]. Proposed mechanisms include cytokine-driven extra-thyroidal PCT synthesis during sterile inflammation, tissue injury-related immune activation, and a contributory effect of renal dysfunction on circulating PCT concentrations [21,24,27].

Clinically, these findings do not suggest that PCT should be ignored in patients with rhabdomyolysis. Rather, PCT should not be interpreted in isolation. In patients with severe muscle injury and renal dysfunction, elevated PCT may reflect sterile tissue injury, impaired renal handling, infection, or a combination of these factors. Therefore, antimicrobial decisions should be based on the full clinical picture, including hemodynamic status, fever trajectory, localizing symptoms, radiological findings, microbiological cultures, urinalysis, serial inflammatory markers, and reassessment over time. In stable patients without convincing clinical, radiological, or microbiological evidence of infection, isolated PCT elevation in the setting of marked rhabdomyolysis should prompt cautious interpretation rather than automatic antibiotic escalation [32]. Conversely, antibiotics should not be withheld when clinical evidence supports infection or sepsis.

This study has several limitations. First, the sample size of 39 patients was modest, limiting statistical power and subgroup analyses. No formal a priori power calculation was performed because the study was designed as an exploratory prospective observational analysis of consecutively enrolled patients at a single centre. Therefore, the absence of a statistically significant independent association between infection status and PCT should not be interpreted as definitive evidence of no effect, but rather as an inconclusive finding in a cohort at risk of Type II error. Similar cohort sizes have been reported in other studies of non-traumatic or mixed-etiology rhabdomyolysis, reflecting the rarity of well-documented cases and the practical challenges of patient enrollment [2,8,9,15,40].

Second, the cohort was predominantly male and restricted to intoxication- or immobilization-related “found-down” rhabdomyolysis. Therefore, extrapolation to female patients and to other rhabdomyolysis etiologies should be made cautiously. Third, some misclassification of infection status cannot be entirely excluded despite predefined diagnostic criteria, blinded adjudication, and consensus review. Occult, early, or low-grade infections may have remained undetected, and no standardized prospective infection-surveillance protocol beyond routine clinical evaluation was performed. The single-centre setting may also limit external validity.

Fourth, total CK was used as the principal biochemical marker of muscle injury. Although CK is widely used in rhabdomyolysis, it is not completely specific to skeletal muscle and may also increase with myocardial injury. Patients with clinically diagnosed myocardial infarction were excluded, and the marked elevation of admission myoglobin supported extensive skeletal muscle injury [41,42]. However, CK-MB, cardiac troponin, and CK-MB/total CK ratios were not systematically available for all patients; therefore, occult cardiac injury cannot be fully excluded as a potential confounder. Fifth, although none of the enrolled patients had documented chronic kidney disease before admission, undiagnosed chronic renal impairment or unavailable pre-admission renal function data cannot be completely excluded.

Finally, several admission regression models showed residual non-normality and evidence of heteroscedasticity, reflecting the markedly skewed distribution of PCT and related biomarkers. In addition, CK, myoglobin, CRP, creatinine, and urea captured overlapping aspects of muscle injury, systemic inflammation, and renal dysfunction, limiting the interpretability of models containing several biologically related predictors. Heteroscedasticity-robust standard errors and bootstrap bias-corrected and accelerated confidence intervals were used to improve robustness, but these methods cannot fully overcome the limitations associated with modest sample size and potential model instability. The exploratory CK × hospital day interaction should also be interpreted cautiously because the number of repeated observations varied between patients.

## 5. Conclusions

In this exploratory single-centre cohort of patients with intoxication- or immobilization-related non-traumatic rhabdomyolysis, PCT elevation was associated with renal dysfunction and markers of muscle injury. Creatinine was the most robust admission predictor, while CK was associated with PCT in the primary admission model, microbiology-only sensitivity model, and longitudinal analyses, but not in the broader admission sensitivity model including overlapping inflammatory and muscle-injury markers. Infection status was not independently associated with PCT in the fitted models; however, the modest sample size and potential infection-classification uncertainty mean that an infection-related contribution cannot be excluded. PCT should therefore be interpreted cautiously in rhabdomyolysis and integrated with clinical, microbiological, and radiological evidence rather than used as an isolated trigger for antimicrobial escalation. Larger prospective studies are needed to clarify the independent contributions of muscle injury, renal dysfunction, and infection to PCT elevation in different rhabdomyolysis etiologies.

## Figures and Tables

**Table 1:** Baseline characteristics of patients with non-traumatic RM stratified by infection status.

Variable	Full Cohort (n = 39)	Not Infected (n = 20)	Infected (n = 19)	*p*-Value
**Demographics**				
Age, years (mean ± SD)	47.8 ± 17.1	42.5 ± 12.8	53.4 ± 19.5	0.043
Male sex, n (%)	31 (79.5)	18 (90.0)	13 (68.4)	0.127 *
Female sex, n (%)	8 (20.5)	2 (10.0)	6 (31.6)	
**Microbiological confirmation**				
Microbiologically proven infection, n (%)	10 (25.6)	0 (0.0)	10 (52.6)	—†
**Laboratory values (median [IQR])**				
Procalcitonin, ng/mL	1.83 [0.175–5.11]	1.60 [0.193–4.07]	1.83 [0.228–6.84]	0.779
Creatine kinase, U/L	11,422 [3463–28,995]	9860 [4794–30,431]	11,436 [1161–27,627]	0.607
Myoglobin, µg/L	6758 [1769–27,079]	6371 [1550–18,410]	8554 [2140–30,000]	0.316
C-reactive protein, mg/L	24.5 [4.50–49.7]	13.4 [2.79–35.5]	40.9 [8.81–126]	0.079
Creatinine, µmol/L	140 [88.0–295]	118 [89.7–302]	228 [83.7–283]	0.593
Urea, mmol/L	8.64 [6.65–13.2]	7.46 [5.93–10.2]	9.93 [7.66–14.6]	0.142

IQR, interquartile range. * Fisher’s exact test. † Shown descriptively only; no *p*-value reported because microbiological confirmation is nested within the infected group definition.

**Table 2:** Selected Spearman correlations between admission biomarkers and age.

Variable 1	Variable 2	Spearman’s ρ	Adjusted *p*-Value
Urea	Creatinine	0.541	0.004
Urea	CK	0.34	0.082
Urea	CRP	0.528	0.004
Urea	PCT	0.528	0.004
Creatinine	PCT	0.587	<0.001
Creatinine	Age	−0.332	0.082
CK	Myoglobin	0.736	<0.001
CK	CRP	0.393	0.036
CK	PCT	0.486	0.005
Myoglobin	PCT	0.326	0.082
CRP	PCT	0.501	0.004

Sample size note: for most pairs, n = 39; for correlations involving urea, n = 37; *p*-values were adjusted for multiple comparisons using the Benjamini–Hochberg false-discovery-rate method across all 21 tested pairwise correlations. Significance was interpreted according to adjusted *p*-values.

## Data Availability

The original contributions presented in the study are included in the article material, and further inquiries can be directed to the corresponding authors.

## Appendix A

Below are the figures for the primary admission regression model, which included PCT (log10) as the dependent variable and CK (log10), creatinine (log10), and infection status as predictors.
